# The Age-Dependent Relationship Between Vascular Risk Factors and Trajectories of Depressed Mood

**DOI:** 10.1093/geroni/igaa057.3266

**Published:** 2020-12-16

**Authors:** Maria Blöchl, Lina Schaare, Ute Kunzmann, Steffen Nestler

**Affiliations:** 1 University of Münster, Leipzig, Germany; 2 Max Planck Institute for Human Cognitive and Brain Sciences, Leipzig, Germany; 3 University of Leipzig, Leipzig, Sachsen, Germany; 4 University of Münster, Münster, Nordrhein-Westfalen, Germany

## Abstract

Cardiovascular risk factors (CVRFs) have been linked to depression, but it is still unclear whether this association becomes stronger or weaker from mid- to later life. Thus, our main aim was to investigate the influence of age on the associations between CRVFs and trajectories of depressed mood. Our sample included 6835 individuals (aged 52–89 years) from the English Longitudinal Study of Ageing (ELSA), who were free of manifest vascular disease at baseline and had bi-yearly measurements of depressed mood over ten years. A composite score incorporated the presence of five CVRFs: hypertension, diabetes, smoking, obesity, and hypercholesterolemia. We used second-order latent growth models to examine the effect of CVRFs, age, and their interaction on levels and changes in depressed mood over time. Our results revealed that baseline CVRFs were associated with higher levels of depressed mood. This association decreased with age and was stronger in midlife compared to later life. CVRFs were not related to changes in depressed mood, indicating that these differences remained stable over time. These findings suggest that CVRFs in midlife, but less so in older age, predict stable differences in depressed mood. They are consistent with reports on the importance of CVRFs in midlife and may support the idea that prevention of vascular burden in this age period may be critical to maintain mental health.

